# Supplementary material for: Design of multi-epitope vaccine candidate against Brucella type IV secretion system (T4SS)
Source: PLoS One. 2023 Aug 10;18(8):e0286358. doi: 10.1371/journal.pone.0286358 (PMC10414599; doi:10.1371/journal.pone.0286358)
Supplement: S1 Table — (DOCX) [file pone.0286358.s001.docx]

| **S1 Table. MHC-I Binding Prediction Results of VirB8(IEDB)** | | | | | |
| --- | --- | --- | --- | --- | --- |
| Allele | start | end | peptide | Score | Percentile Rank |
| HLA-A*11:01 | 95 | 104 | SVSYDTVMDK | 0.805032 | 0.08 |
| HLA-A*11:01 | 164 | 173 | TIVSIVPNGK | 0.394412 | 0.43 |
| HLA-A*11:01 | 96 | 105 | VSYDTVMDKY | 0.253758 | 0.7 |
| HLA-A*11:01 | 4 | 13 | RKQSPQKSVK | 0.078159 | 1.6 |
| HLA-A*11:01 | 30 | 39 | EAAHVRLVEK | 0.052614 | 1.6 |
| HLA-A*02:01 | 217 | 226 | RLTNPLGFNV | 0.268047 | 0.61 |
| HLA-A*02:01 | 63 | 72 | AGMLPLKQHV | 0.162002 | 0.97 |
| HLA-A*02:01 | 69 | 78 | KQHVPYLVRV | 0.129666 | 1.2 |
| HLA-A*02:01 | 150 | 159 | ALDKQYGSNV | 0.083768 | 1.6 |
| HLA-A*02:01 | 87 | 96 | ILTSLDEKSV | 0.081068 | 1.6 |
| HLA-A*03:01 | 95 | 104 | SVSYDTVMDK | 0.793266 | 0.09 |
| HLA-A*03:01 | 164 | 173 | TIVSIVPNGK | 0.341584 | 0.58 |
| HLA-A*03:01 | 4 | 13 | RKQSPQKSVK | 0.26425 | 0.74 |
| HLA-A*03:01 | 96 | 105 | VSYDTVMDKY | 0.218995 | 0.88 |
| HLA-A*03:01 | 26 | 35 | ALNWEAAHVR | 0.102619 | 1.5 |
