# Supplementary material for: Design of multi-epitope vaccine candidate against Brucella type IV secretion system (T4SS)
Source: PLoS One. 2023 Aug 10;18(8):e0286358. doi: 10.1371/journal.pone.0286358 (PMC10414599; doi:10.1371/journal.pone.0286358)
Supplement: S2 Table — (DOCX) [file pone.0286358.s002.docx]

| **S2 Table. MHC-I Binding Prediction Results of VirB8(NetCTLpan version 1.1)** | | | | | |
| --- | --- | --- | --- | --- | --- |
| Allele | start | end | peptide | Score | Percentile Rank |
| HLA-A*11:01 | 115 | 124 | ETYDWYTLQK | 0.95573 | 0.20 |
| HLA-A*11:01 | 95 | 104 | SVSYDTVMDK | 0.89369 | 0.40 |
| HLA-A*11:01 | 176 | 185 | GTVRFAKTTK | 0.88513 | 0.40 |
| HLA-A*11:01 | 164 | 173 | TIVSIVPNGK | 0.80875 | 0.80 |
| HLA-A*11:01 | 195 | 204 | TTHWIATIGY | 0.78743 | 1.00 |
| HLA-A*02:01 | 198 | 207 | WIATIGYQYV | 0.90778 | 0.80 |
| HLA-A*02:01 | 56 | 65 | VLLGIGIAGM | 0.79495 | 1.50 |
| HLA-A*02:01 | 217 | 226 | RLTNPLGFNV | 0.74727 | 1.50 |
| HLA-A*02:01 | 120 | 129 | YTLQKDYETV | 0.70989 | 2.00 |
| HLA-A*02:01 | 150 | 159 | ALDKQYGSNV | 0.64382 | 3.00 |
| HLA-A*03:01 | 115 | 124 | ETYDWYTLQK | 0.72473 | 0.80 |
| HLA-A*03:01 | 176 | 185 | GTVRFAKTTK | 0.67213 | 1.00 |
| HLA-A*03:01 | 95 | 104 | SVSYDTVMDK | 0.66469 | 1.00 |
| HLA-A*03:01 | 195 | 204 | TTHWIATIGY | 0.62343 | 1.50 |
| HLA-A*03:01 | 101 | 110 | VMDKYWLSQY | 0.60927 | 1.50 |
