# Supplementary material for: Design of multi-epitope vaccine candidate against Brucella type IV secretion system (T4SS)
Source: PLoS One. 2023 Aug 10;18(8):e0286358. doi: 10.1371/journal.pone.0286358 (PMC10414599; doi:10.1371/journal.pone.0286358)
Supplement: S3 Table — (DOCX) [file pone.0286358.s003.docx]

| **S3 Table. MHC-I Binding Prediction Results of VirB10(IEDB)** | | | | | |
| --- | --- | --- | --- | --- | --- |
| Allele | start | end | peptide | Score | Percentile Rank |
| HLA-A*11:01 | 346 | 355 | TINIPPTLYK | 0.970237 | 0.01 |
| HLA-A*11:01 | 71 | 80 | TSTVPMRTFK | 0.567391 | 0.23 |
| HLA-A*11:01 | 130 | 139 | SASALMVVTK | 0.499431 | 0.3 |
| HLA-A*11:01 | 335 | 344 | TSNLASTALK | 0.485712 | 0.31 |
| HLA-A*11:01 | 155 | 164 | ALLDSQKNTK | 0.458155 | 0.35 |
| HLA-A*02:01 | 79 | 88 | FKLPPPPPPA | 0.536512 | 0.24 |
| HLA-A*02:01 | 185 | 194 | SLLRNRDFLL | 0.23751 | 0.68 |
| HLA-A*02:01 | 207 | 216 | RLDSTVPGMA | 0.105932 | 1.3 |
| HLA-A*02:01 | 203 | 214 | ALQTRLDSTV | 0.087667 | 1.5 |
| HLA-A*02:01 | 206 | 215 | TRLDSTVPGM | 0.084726 | 1.5 |
| HLA-A*03:01 | 346 | 355 | TINIPPTLYK | 0.964238 | 0.01 |
| HLA-A*03:01 | 155 | 164 | ALLDSQKNTK | 0.748604 | 0.13 |
| HLA-A*03:01 | 152 | 161 | RIQALLDSQK | 0.702853 | 0.16 |
| HLA-A*03:01 | 254 | 263 | RIYVLWTRVK | 0.620455 | 0.23 |
| HLA-A*03:01 | 71 | 80 | TSTVPMRTFK | 0.315537 | 0.63 |
