# Supplementary material for: Design of multi-epitope vaccine candidate against Brucella type IV secretion system (T4SS)
Source: PLoS One. 2023 Aug 10;18(8):e0286358. doi: 10.1371/journal.pone.0286358 (PMC10414599; doi:10.1371/journal.pone.0286358)
Supplement: S4 Table — (DOCX) [file pone.0286358.s004.docx]

| **S4 Table. MHC-I Binding Prediction Results of VirB10(NetCTLpan version 1.1)** | | | | | |
| --- | --- | --- | --- | --- | --- |
| Allele | start | end | peptide | Score | Percentile Rank |
| HLA-A*11:01 | 346 | 355 | TINIPPTLYK | 1.05250 | 0.05 |
| HLA-A*11:01 | 130 | 139 | SASALMVVTK | 0.90331 | 0.40 |
| HLA-A*11:01 | 71 | 80 | TSTVPMRTFK | 0.90282 | 0.40 |
| HLA-A*11:01 | 335 | 344 | TSNLASTALK | 0.88694 | 0.40 |
| HLA-A*11:01 | 152 | 161 | RIQALLDSQK | 0.82417 | 0.80 |
| HLA-A*02:01 | 36 | 45 | FLFVVGFIVV | 0.94930 | 0.40 |
| HLA-A*02:01 | 300 | 309 | ALMLSTIETL | 0.94066 | 0.80 |
| HLA-A*02:01 | 38 | 47 | FVVGFIVVLL | 0.81710 | 1.00 |
| HLA-A*02:01 | 185 | 194 | SLLRNRDFLL | 0.75675 | 1.50 |
| HLA-A*02:01 | 45 | 54 | VLLLLLVFHM | 0.72134 | 2.00 |
| HLA-A*03:01 | 346 | 355 | TINIPPTLYK | 0.91750 | 0.10 |
| HLA-A*03:01 | 254 | 263 | RIYVLWTRVK | 0.85172 | 0.20 |
| HLA-A*03:01 | 152 | 161 | RIQALLDSQK | 0.78917 | 0.40 |
| HLA-A*03:01 | 155 | 164 | ALLDSQKNTK | 0.74624 | 0.80 |
| HLA-A*03:01 | 335 | 344 | TSNLASTALK | 0.64894 | 1.50 |
