# Supplementary material for: Design of multi-epitope vaccine candidate against Brucella type IV secretion system (T4SS)
Source: PLoS One. 2023 Aug 10;18(8):e0286358. doi: 10.1371/journal.pone.0286358 (PMC10414599; doi:10.1371/journal.pone.0286358)
Supplement: S5 Table — (DOCX) [file pone.0286358.s005.docx]

| **S5 Table. MHC-Ⅱ Binding Prediction Results of VirB8(IEDB)** | | | | | |
| --- | --- | --- | --- | --- | --- |
| Allele | start | end | peptide | Score | Percentile Rank |
| HLA-DRB1*03:01 | 30 | 44 | EAAHVRLVEKSERRA | 0.5797 | 2.1 |
| HLA-DRB1*03:01 | 92 | 106 | DEKSVSYDTVMDKYW | 0.5551 | 2.3 |
| HLA-DRB1*03:01 | 31 | 45 | AAHVRLVEKSERRAW | 0.5347 | 2.4 |
| HLA-DRB1*03:01 | 142 | 156 | ASQFQGDKALDKQYG | 0.5354 | 2.4 |
| HLA-DRB1*03:01 | 91 | 105 | LDEKSVSYDTVMDKY | 0.5017 | 2.6 |
| HLA-DRB1*07:01 | 201 | 215 | TIGYQYVNPSLMSES | 0.8026 | 0.46 |
| HLA-DRB1*07:01 | 200 | 214 | ATIGYQYVNPSLMSE | 0.7787 | 0.61 |
| HLA-DRB1*07:01 | 195 | 209 | TTHWIATIGYQYVNP | 0.7284 | 0.88 |
| HLA-DRB1*07:01 | 26 | 40 | ALNWEAAHVRLVEKS | 0.7013 | 0.97 |
| HLA-DRB1*07:01 | 25 | 39 | EALNWEAAHVRLVEK | 0.6677 | 1.2 |
| HLA-DRB1*15:01 | 65 | 79 | MLPLKQHVPYLVRVN | 0.3525 | 4 |
| HLA-DRB1*15:01 | 72 | 86 | VPYLVRVNAQTGAPD | 0.3352 | 4.2 |
| HLA-DRB1*15:01 | 163 | 177 | VTIVSIVPNGKGIGT | 0.3087 | 4.8 |
| HLA-DRB1*15:01 | 64 | 80 | GMLPLKQHVPYLVRV | 0.2704 | 5.7 |
| HLA-DRB1*15:01 | 71 | 85 | HVPYLVRVNAQTGAP | 0.2593 | 5.9 |
