# Supplementary material for: Design of multi-epitope vaccine candidate against Brucella type IV secretion system (T4SS)
Source: PLoS One. 2023 Aug 10;18(8):e0286358. doi: 10.1371/journal.pone.0286358 (PMC10414599; doi:10.1371/journal.pone.0286358)
Supplement: S6 Table — (DOCX) [file pone.0286358.s006.docx]

| **S6 Table. MHC-Ⅱ Binding Prediction Results of VirB8(NetMHCIIpan version 4.0)** | | | | | |
| --- | --- | --- | --- | --- | --- |
| Allele | start | end | peptide | Score | Percentile Rank |
| HLA-DRB1*03:01 | 225 | 239 | NVTSYRVDPEMGVVQ | 0.649903 | 1.22 |
| HLA-DRB1*03:01 | 92 | 106 | DEKSVSYDTVMDKYW | 0.572403 | 1.62 |
| HLA-DRB1*03:01 | 142 | 156 | ASQFQGDKALDKQYG | 0.563436 | 1.66 |
| HLA-DRB1*03:01 | 30 | 44 | EAAHVRLVEKSERRA | 0.544387 | 1.76 |
| HLA-DRB1*03:01 | 141 | 155 | YASQFQGDKALDKQY | 0.526181 | 1.87 |
| HLA-DRB1*07:01 | 201 | 215 | TIGYQYVNPSLMSES | 0.825272 | 0.34 |
| HLA-DRB1*07:01 | 200 | 214 | ATIGYQYVNPSLMSE | 0.800641 | 0.40 |
| HLA-DRB1*07:01 | 195 | 209 | TTHWIATIGYQYVNP | 0.711571 | 0.67 |
| HLA-DRB1*07:01 | 199 | 203 | IATIGYQYVNPSLMS | 0.667533 | 0.84 |
| HLA-DRB1*07:01 | 26 | 40 | ALNWEAAHVRLVEKS | 0.618687 | 1.06 |
| HLA-DRB1*15:01 | 163 | 177 | VTIVSIVPNGKGIGT | 0.299462 | 3.31 |
| HLA-DRB1*15:01 | 162 | 176 | SVTIVSIVPNGKGIG | 0.267165 | 3.73 |
| HLA-DRB1*15:01 | 72 | 86 | VPYLVRVNAQTGAPD | 0.184721 | 5.37 |
| HLA-DRB1*15:01 | 65 | 79 | MLPLKQHVPYLVRVN | 0.175506 | 5.62 |
| HLA-DRB1*15:01 | 223 | 237 | GFNVTSYRVDPEMGV | 0.134734 | 6.98 |
