# Supplementary material for: Design of multi-epitope vaccine candidate against Brucella type IV secretion system (T4SS)
Source: PLoS One. 2023 Aug 10;18(8):e0286358. doi: 10.1371/journal.pone.0286358 (PMC10414599; doi:10.1371/journal.pone.0286358)
Supplement: S7 Table — (DOCX) [file pone.0286358.s007.docx]

| **S7 Table. MHC-Ⅱ Binding Prediction Results of VirB10(IEDB)** | | | | | |
| --- | --- | --- | --- | --- | --- |
| Allele | start | end | peptide | Score | Percentile Rank |
| HLA-DRB1*03:01 | 265 | 279 | PNGVVIDLDSPGADP | 0.7914 | 0.89 |
| HLA-DRB1*03:01 | 264 | 278 | TPNGVVIDLDSPGAD | 0.7251 | 1.2 |
| HLA-DRB1*03:01 | 266 | 280 | NGVVIDLDSPGADPL | 0.6356 | 1.7 |
| HLA-DRB1*03:01 | 263 | 277 | KTPNGVVIDLDSPGA | 0.5829 | 2.1 |
| HLA-DRB1*03:01 | 267 | 281 | GVVIDLDSPGADPLG | 0.4561 | 3 |
| HLA-DRB1*07:01 | 141 | 155 | SGDTVVQTTNARIQA | 0.9487 | 0.06 |
| HLA-DRB1*07:01 | 142 | 156 | GDTVVQTTNARIQAL | 0.9389 | 0.08 |
| HLA-DRB1*07:01 | 140 | 154 | SSGDTVVQTTNARIQ | 0.9259 | 0.11 |
| HLA-DRB1*07:01 | 143 | 157 | DTVVQTTNARIQALL | 0.8154 | 0.43 |
| HLA-DRB1*07:01 | 187 | 201 | LRNRDFLLAKGSIIN | 0.8119 | 0.44 |
| HLA-DRB1*15:01 | 306 | 320 | IETLGRYATQKVGGG | 0.9514 | 0.03 |
| HLA-DRB1*15:01 | 305 | 319 | TIETLGRYATQKVGG | 0.9275 | 0.07 |
| HLA-DRB1*15:01 | 307 | 321 | ETLGRYATQKVGGGG | 0.8939 | 0.14 |
| HLA-DRB1*15:01 | 304 | 318 | STIETLGRYATQKVG | 0.8168 | 0.51 |
| HLA-DRB1*15:01 | 142 | 156 | GDTVVQTTNARIQAL | 0.5114 | 2 |
