# Supplementary material for: Design of multi-epitope vaccine candidate against Brucella type IV secretion system (T4SS)
Source: PLoS One. 2023 Aug 10;18(8):e0286358. doi: 10.1371/journal.pone.0286358 (PMC10414599; doi:10.1371/journal.pone.0286358)
Supplement: S8 Table — (DOCX) [file pone.0286358.s008.docx]

| **S8 Table. MHC-Ⅱ Binding Prediction Results of VirB10(NetMHCIIpan version 4.0)** | | | | | |
| --- | --- | --- | --- | --- | --- |
| Allele | start | end | peptide | Score | Percentile Rank |
| HLA-DRB1*03:01 | 265 | 279 | PNGVVIDLDSPGADP | 0.743127 | 0.81 |
| HLA-DRB1*03:01 | 264 | 278 | TPNGVVIDLDSPGAD | 0.668810 | 1.13 |
| HLA-DRB1*03:01 | 266 | 280 | NGVVIDLDSPGADPL | 0.602979 | 1.45 |
| HLA-DRB1*03:01 | 263 | 277 | KTPNGVVIDLDSPGA | 0.540577 | 1.78 |
| HLA-DRB1*03:01 | 267 | 281 | GVVIDLDSPGADPLG | 0.434762 | 2.51 |
| HLA-DRB1*07:01 | 141 | 155 | SGDTVVQTTNARIQA | 0.918911 | 0.12 |
| HLA-DRB1*07:01 | 142 | 156 | GDTVVQTTNARIQAL | 0.894987 | 0.17 |
| HLA-DRB1*07:01 | 140 | 154 | SSGDTVVQTTNARIQ | 0.881985 | 0.20 |
| HLA-DRB1*07:01 | 187 | 201 | LRNRDFLLAKGSIIN | 0.881985 | 0.20 |
| HLA-DRB1*07:01 | 64 | 78 | SYKTMVQTSTVPMRT | 0.781261 | 0.45 |
| HLA-DRB1*15:01 | 358 | 372 | GEEIGIYIARDLDFS | 0.944781 | 0.12 |
| HLA-DRB1*15:01 | 357 | 371 | QGEEIGIYIARDLDF | 0.930821 | 0.16 |
| HLA-DRB1*15:01 | 306 | 320 | IETLGRYATQKVGGG | 0.927006 | 0.17 |
| HLA-DRB1*15:01 | 356 | 370 | NQGEEIGIYIARDLD | 0.907509 | 0.23 |
| HLA-DRB1*15:01 | 305 | 319 | TIETLGRYATQKVGG | 0.894463 | 0.26 |
