# Supplementary material for: Design of multi-epitope vaccine candidate against Brucella type IV secretion system (T4SS)
Source: PLoS One. 2023 Aug 10;18(8):e0286358. doi: 10.1371/journal.pone.0286358 (PMC10414599; doi:10.1371/journal.pone.0286358)
Supplement: S9 Table — (DOCX) [file pone.0286358.s009.docx]

| **S9 Table. LBEs Results of VirB8 and VirB10(ABCpred)** | | | | |
| --- | --- | --- | --- | --- |
|  | start | end | peptide | Rank |
| VirB8 | 28 | 47 | NWEAAHVRLVEKSERRAWKI | 1 |
|  | 18 | 37 | NAPSVYDEALNWEAAHVRLV | 2 |
|  | 8 | 27 | PQKSVKNGQGNAPSVYDEAL | 3 |
|  | 72 | 91 | VPYLVRVNAQTGAPDILTSL | 4 |
| VirB10 | 99 | 118 | PAMPIAEPAAAALSLPPLPD | 1 |
|  | 329 | 348 | TGGGESTSNLASTALKDTIN | 2 |
|  | 111 | 130 | LSLPPLPDDTPAKDDVLDKS | 3 |
|  | 11 | 30 | GTLDGERGLPTVNENGSGRT | 4 |
