# Supplementary figures and images for: Design of multi-epitope vaccine candidate against Brucella type IV secretion system (T4SS)
Source: PLoS One. 2023 Aug 10;18(8):e0286358. doi: 10.1371/journal.pone.0286358 (PMC10414599; doi:10.1371/journal.pone.0286358)

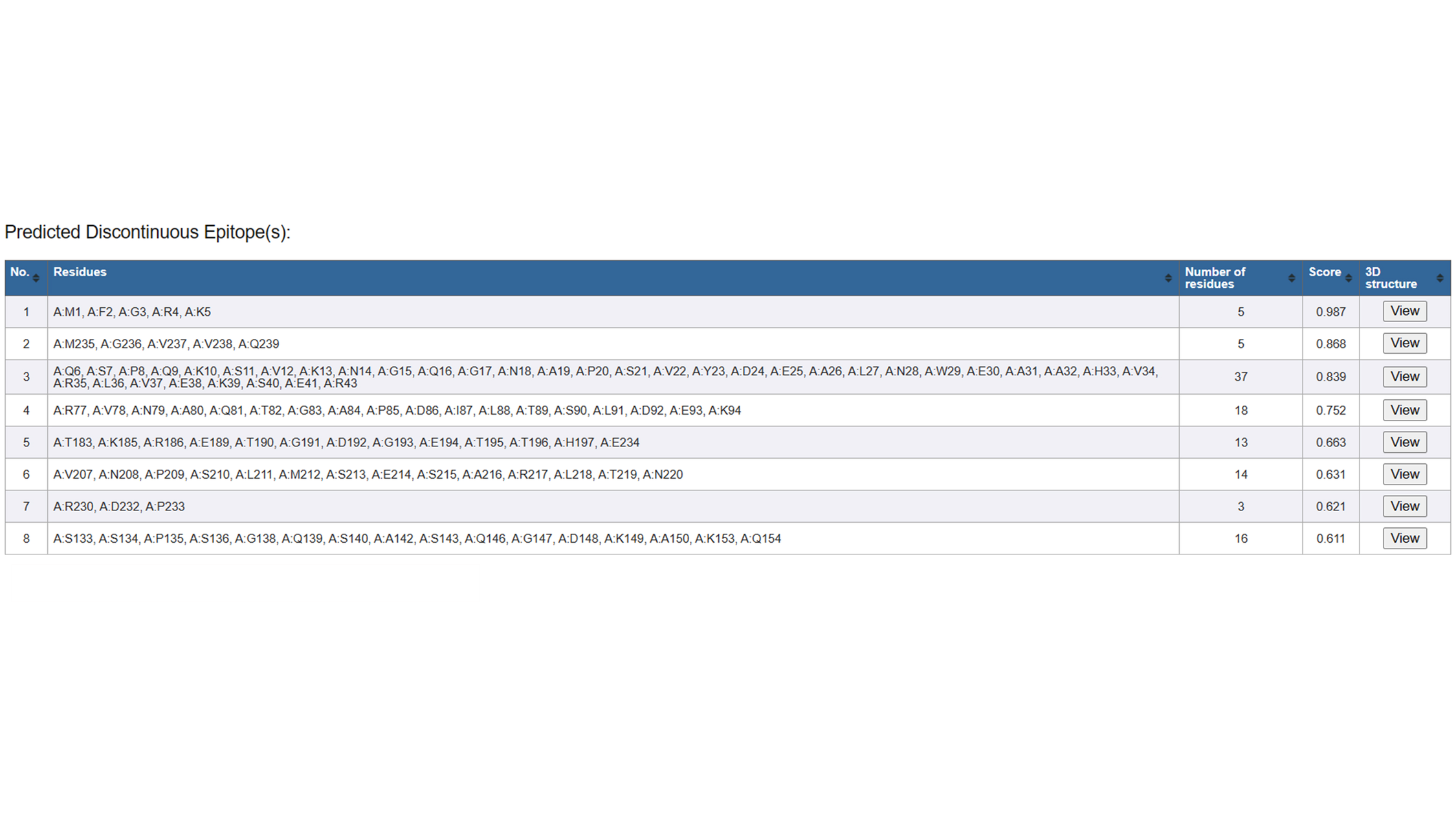

Supplement: S1 Fig — (TIF) [file pone.0286358.s010.tif]

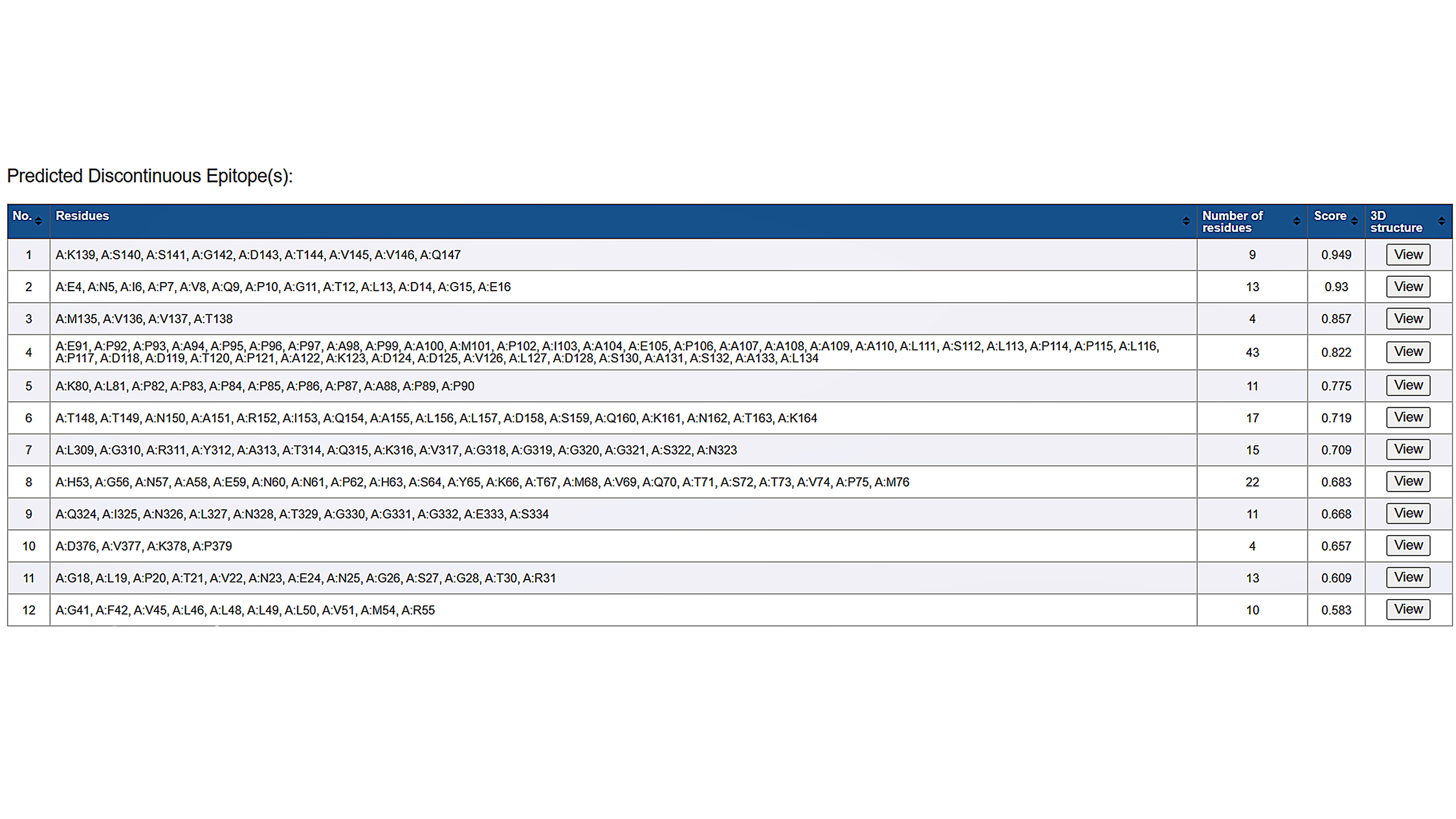

Supplement: S2 Fig — (TIF) [file pone.0286358.s011.tif]
