# Supplementary material for: Design of multi-epitope vaccine candidate against Brucella type IV secretion system (T4SS)
Source: PLoS One. 2023 Aug 10;18(8):e0286358. doi: 10.1371/journal.pone.0286358 (PMC10414599; doi:10.1371/journal.pone.0286358)
Supplement: Attachment — Submitted filename: PONE-D-23-13551 comments.docx [file pone.0286358.s013.docx]

The authors have done tremendous work entitled "**Design of multiepitope vaccine candidate against Brucella type Ⅳ secretion system（T4SS)**" in the field of vaccine development. There is space to improve the manuscript to be published in this prestigious journal. Here are some suggestions which will improve the quality of the manuscript.

**Comments**

1. The abstract should start with the introduction of the bacteria that the authors selected. For a clear understanding, add two introduction lines to the bacteria in the abstract.
2. The overall abstract is in the form of methodology. What promising results about the vaccine or epitopes the authors get are lacking in the abstract. The abstract should be modified according to the results and a clear conclusion.
3. The aim of the study should be discussed, unlike the methodology in the introduction. For better understanding, provide a clear study goal of why the vaccine is essential for the selected bacteria.
4. In the "1.1 Material sources" section, the authors retrieve the sequences from the UniProt database, but no ID is mentioned of any of the sequences, and also provide the UniProt database link.
5. In the section "1.2.1 Selection of target proteins", the authors find the antigenicity only for the selected proteins. First, discuss which proteins are selected from the pathogen and calculate the allergenicity of the proteins.
6. The sequence search and material sources sections should be combined in one subtopic and presented at the start of the methodology.
7. In the "Prediction of protein T-cell epitopes" section, the authors took six alleles for CTL prediction and three alleles for HTL prediction. Provide the population coverage analysis of these alleles to justify the selection of these alleles.
8. Explain how the final epitopes were selected from the prediction in the "Prediction of protein T-cell epitopes" section.
9. Similarly, in the B-cell predictions elaborates the process of final epitopes selection from the prediction.
10. The selected epitopes' physiochemical properties, antigenicity and allergenicity, should be calculated and reported as a safe selection for the Multiepitope vaccines.
11. The authors used the MEV-TLR4 complex in section "1.2.15 Molecular dynamics simulation", but did not mention any docking protocols or details of the Multiepitope vaccine and TLR4 receptor.
12. The section "1.2.15 Molecular dynamics simulation" is very poorly explained. The simulation protocols used by the server should be explained in detail, like which forcefields are used and what other parameters are provided to the simulation.
13. The authors stated in lines 210-211 that "the deformability, stiffness and stability of the complex with the output results". What analysis they performed after the simulation for what purpose should be adequately explained, like RMSD, RMSF, Radius of gyration, hydrogen bond analysis, PCA, etc.
14. The figure captions should be with the figure to explain it adequately, not in the manuscript's text.
15. The section "2.10 Quality assessment of models" should be appropriately discussed. Provide all the details of how much the favoured region is, allowed and disallowed regions of the model in the text.
16. The section "2.11 Molecular docking" is very poorly explained. Explain how many hydrogen bonds are formed between the MEV and TLR4 and how many salt bridges are formed.
17. The authors select the monomer of the TLR4 receptor, but its active form is the dimmer. Is there any specific reason for the monomer selection? Explain it, please, in the methodology.
18. The authors stated in lines 397-399 that "The eigenvalue in the eigenvalue plot is 9.353365e-05, which, according to previous studies, indicates that the complex has low deformability and good stability" but did not provide any sufficient references to the sentence.
19. The selection "2.15 Molecular dynamics simulation" results are discussed very poorly. Kindly search for some literature on how to explain these results in a better way. The output of the results is explained like a methodology. Focus on what good results you obtained from the output and discuss those just.
20. Add vaccine development studies for the selected pathogen done before in the discussion section and compare with your outputs.
